# Pretreatment with coenzyme Q10 improves ovarian response and embryo quality in low-prognosis young women with decreased ovarian reserve: a randomized controlled trial

**DOI:** 10.1186/s12958-018-0343-0

**Published:** 2018-03-27

**Authors:** Yangying Xu, Victoria Nisenblat, Cuiling Lu, Rong Li, Jie Qiao, Xiumei Zhen, Shuyu Wang

**Affiliations:** 1 Department of Reproduction, Beijing Obstetrics and Gynecology Hospital, Capital Medical University, Beijing, 100026 China; 2 Reproductive Medical Center, Department of Obstetrics and Gynecology, Peking University Third Hospital, Beijing, 100123 China; 3 Key Laboratory of Assisted Reproduction, Ministry of Education, Beijing, China

**Keywords:** Poor ovarian response, POSEIDON stratification, Oxidative stress, Coenzyme Q10, In vitro fertilization, High-quality embryos, Clinical outcomes

## Abstract

**Background:**

Management of women with reduced ovarian reserve or poor ovarian response (POR) to stimulation is one of the major challenges in reproductive medicine. The primary causes of POR remain elusive and oxidative stress was proposed as one of the important contributors. It has been suggested that focus on the specific subpopulations within heterogeneous group of poor responders could assist in evaluating optimal management strategies for these patients. This study investigated the effect of anti-oxidant treatment with coenzyme Q10 (CoQ10) on ovarian response and embryo quality in young low-prognosis patients with POR.

**Methods:**

This prospective, randomized controlled study included 186 consecutive patients with POR stratified according to the POSEIDON classification group 3 (age < 35, poor ovarian reserve parameters). The participants were randomized to the CoQ10 pre-treatment for 60 days preceding IVF-ICSI cycle or no pre-treatment. The number of high quality embryos was a primary outcome measure.

**Results:**

A total of 169 participants were evaluated (76 treated with CoQ10 and 93 controls); 17 women were excluded due to low compliance with CoQ10 administration. The baseline demographic and clinical characteristics were comparable between the groups. CoQ10 pretreatment resulted in significantly lower gonadotrophin requirements and higher peak E2 levels. Women in CoQ10 group had increased number of retrieved oocytes (4, IQR 2–5), higher fertilization rate (67.49%) and more high-quality embryos (1, IQR 0–2); *p* < 0.05. Significantly less women treated with CoQ10 had cancelled embryo transfer because of poor embryo development than controls (8.33% vs. 22.89%, *p* = 0.04) and more women from treatment group had available cryopreserved embryos (18.42% vs. 4.3%, *p* = 0.012). The clinical pregnancy and live birth rates per embryo transfer and per one complete stimulation cycle tended to be higher in CoQ10 group but did not achieve statistical significance.

**Conclusion:**

Pretreatment with CoQ10 improves ovarian response to stimulation and embryological parameters in young women with poor ovarian reserve in IVF-ICSI cycles. Further work is required to determine whether there is an effect on clinical treatment endpoints.

## Background

Poor response to controlled ovarian hyperstimulation (COH) remains one of the main challenges of the assisted reproductive technology (ART) treatments. Despite impressive advances in the field, many women exhibit inadequate response to gonadotrophins, referred to as ‘poor or low responders’ and have higher odds of cycle cancellation, fewer oocytes at retrieval, lower oocyte quality and reduced number of embryos for transfer. Collectively, this results in serial failure of the ART cycles and is frustrating for both patients and their caregivers. The exact incidence of the condition is hard to establish owing to variable definitions in literature with the estimates ranging from 5.6 to 35.1% of ART cycles [1]. Multiple interventions have been proposed to improve reproductive outcomes in women with poor ovarian response (POR), but the randomized intervention studies and meta-analyses of these studies reveal conflicting results [2, 3]. Currently, the evidence-based therapeutic strategies to improve ovarian response and reproductive outcomes in women with POR are lacking, and treating clinicians often offer empirical treatments with little clinical evidence to support their use [4]. Furthermore, it has been increasingly acknowledged that the available ovarian reserve tests are not reliable to predict pregnancy after assisted conception [5]. We do not have universally accepted tests to predict response to treatment, which is of important value for counseling couples regarding their treatment pathways and for setting patients’ expectations.

It has been proposed that a heterogeneity of the included population is the main barrier in evaluating the interventions and the factors that guide prognosis for POR [6]. An internationally-agreed consensus on the definition of POR reached by an ESHRE Campus Workshop held in Bologna in 2010 suggests that at least 2 out of 3 features must be present: (1) advanced maternal age or any other risk factor for POR; (2) previous POR; (3) abnormal ovarian reserve test [7]. This uniform definition, however, implies that POR constitutes heterogeneous group of women with respect to age, previous reproductive experience and ovarian reserve tests that may have different response to the interventions [6]. While age-dependent decline in ovarian reserve and oocyte quality accounts for poor response in older women, an underlying etiology for its occurrence earlier in life is less clear. It is possible that younger women with compromised ovarian reserve represent a distinct subpopulation within POR group, and their fertility prognosis may differ from that of older women with low ovarian reserve markers or from similar age women with adequate ovarian reserve parameters but suboptimal response to ovarian stimulation [8]. 

Taking the above considerations into account, the recently established POSEIDON group (Patient-Oriented Strategies Encompassing Individualize Oocyte Number) proposed a new stratification of women with POR undergoing ART treatments, which includes 4 subgroups based on women’s age, ovarian reserve parameters and previous response to ovarian stimulation [9]. The POSEIDON concept introduces personalized medicine approach to the POR population and is expected to be more effective in identifying the subsets of patients who could benefit from specific interventions [10].

The physiology of poor ovarian response is not fully understood and the molecular events underlying POR remain unknown. Oxidative stress and mitochondrial dysfunction are among the most investigated possible mechanisms [11]. Mitochondria are the most abundant organelles in oocytes and early embryos that generate approximately 90% of reactive oxygen species (ROS), the end products of oxygen metabolism, and convert ROS into an inactive state via antioxidant defense mechanisms [12]. Higher levels of ROS accumulating in mitochondria during multiple physiological conditions contribute to mitochondrial dysfunction and increase in oxidative stress. This, in turn, leads to oxidative damage to DNA and other intra-cellular aberrations, which are similar to the age-related changes [12, 13]. Thus, improving mitochondrial function by supplementing antioxidants has been proposed as one of the important strategies to enhance reproductive performance [11, 14].

Coenzyme Q10 (CoQ10) is a lipid-soluble coenzyme and is an essential component of the inner mitochondrial membrane. CoQ10 is primarily involved in electron transport in the mitochondrial respiratory chain and oxidative phosphorylation to produce adenosine triphosphate (ATP). CoQ10 acts as an antioxidant by inhibiting lipid peroxidation and DNA oxidation, thus is capable of strengthening endogenous antioxidant system within a cell [15]. CoQ10 supplementation has been shown to improve cardiovascular function and male fertility [16–18]. Reduced concentrations of CoQ10 in plasma have been associated with hypogonadism and altered levels of other steroid hormones [19]. Decrease in CoQ10 level is commonly observed in individuals in late 30th and appears to co-occur with the age-related decline in fertility and increased rate of embryo aneuploidy, suggesting a contribution of the reduced expression of CoQ10 to ovarian ageing [20]. Several animal studies have demonstrated that CoQ10 protects ovarian reserve, counteracts physiological ovarian ageing by restoring mitochondrial function and increases the rate of embryo cleavage and blastocyst formation [21–23]. In the clinical setting, CoQ10 supplementation led to better response to ovulation induction and decreased odds of fetal aneuploidy in 35–43-year-old women [24, 25]. To date, however, no study has investigated whether CoQ10 pretreatment could improve the ART treatment outcomes in young subpopulation of poor responders in a randomized setting.

On the above evidence, this study focused on investigating the effect of CoQ10 supplementation on response to ovarian stimulation in the group of young women with diminished ovarian reserve, corresponding to the Poseidon’s stratification group 3 [9]. We hypothesized that increased oxidative stress has a prominent effect on premature decline of ovarian function in these women, which could be amenable to anti-oxidant therapy.

## Methods

### Study design and randomization

**Fig. 1:**
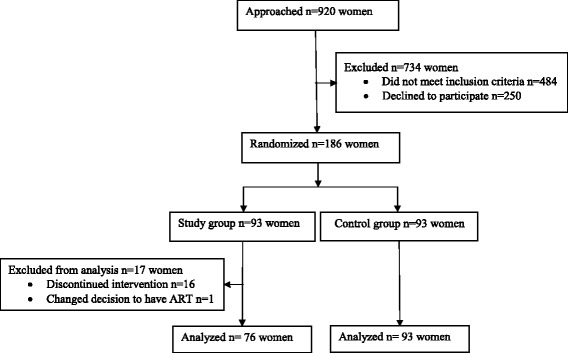
Flow of the patients through the trial

This was a prospective randomized controlled study, conducted at the Reproductive Medical Center of the Peking University Third Hospital, a tertiary university hospital and a center of excellence in Reproductive Medicine in China. The study is reported according to the CONSORT guidelines. The flow of the patients in this study is presented in Fig. 1.

All the participants were randomized 1:1 to either CoQ10 treatment (study group) or no treatment (control group) followed by an ART cycle. The randomization was performed over the period of 14 months (between June 2, 2015 and July 31, 2016) by using the computer-generated randomization codes, which were then placed in the sealed, opaque sequentially numbered envelopes by a third party (nurse practitioner) who was not directly involved in the patient management or in the randomization process. The envelopes were handed out to the participants upon completing the informed consent. The study participants and the investigators were not blinded to the patient grouping. The participants were followed through one completed ART cycle until all frozen embryos generated from the index cycle were used or until delivery in those who achieved pregnancy.

### Study population

All consecutive women who were found to have POR and were referred to IVF-ET cycle in our institution were approached. POR was defined according to the ESHRE Bologna criteria [7]. The study inclusion criteria were: age < 35 years, anti-Mullerian hormone (AMH) < 1.2 ng/ml, and antral follicle count (AFC) < 5, the parameters that corresponded to a low prognosis group 3 as per the POSEIDON stratification [9]. Exclusion criteria were: age ≥ 35 years, history of ovarian surgery, endocrine or autoimmune disease (e.g. diabetes, thyroid disease or presence of anti-thyroid antibodies or PCOS), chromosomal abnormality, uterine malformations, more than 3 previous IVF cycles, treatment with cholesterol-lowering drugs, previous treatment with anti-oxidants (last 5 years) or known allergy to CoQ10 or ubiquinol (the water-soluble isoform of CoQ10). All the participants completed the questionnaire with demographic, medical and reproductive information and underwent clinical examination, pelvic ultrasound, chromosome analysis, AMH test, reproductive endocrine profile and thyroid studies. All the included women were specifically asked about any previous treatment with anti-oxidants such as CoQ10, ubiquinol, vitamin A, vitamin E, vitamin C, beta-carotene or selenium, including the duration and time of treatment.

### Treatment protocols

The intervention in the study group included oral administration of CoQ10 (GNC Holdings Inc., Pittsburg, PA, USA) 200 mg three times a day, for a period of 60 days in an open label fashion. The ART treatment (in vitro fertilization (IVF) or intracytoplasmic sperm injection (ICSI)) was commenced in the first menstrual cycle upon completion of CoQ10 treatment. The control group commenced ART (IVF or ICSI) after enrollment without any additional treatment.

### Ovarian stimulation and oocyte retrieval

All participants underwent ovarian stimulation with the short GnRH-antagonist protocol. A combination of recombinant follicle stimulating hormone (FSH) (Gonal-F, 225 IU/day, Merck Serono SA Aubonne Branch) and human menopausal gonadotrophin (Menotropins for injection FSH 75 IU: LH 75 IU, 225 IU/day, Livzon Pharmaceutical Group Inc.) in a fixed-dose was started on Day 2 of the menstrual cycle with the option to adjust dose according to response after 4 days of stimulation (Day 6 of menstrual cycle). GnRH antagonist (Cetrorelix 250 μg/day, Merck Serono, Darmstadt, Germany) was started when a leading follicle of 12 mm was achieved. Recombinant human chorionic gonadotrophin (hCG) trigger (Ovidrele 250 μg; Merck Serono S.p.A, Rome, Italy) was administered when at least one follicle was above 18 mm. The cycle was cancelled when there were no follicles with diameter ≥ 14 mm after 8–9 days of gonadotrophin therapy or when peak E2 level was below 250 pmol/l.

Ultrasound-guided transvaginal oocyte retrieval was performed 36–38 h after the trigger injection by using a 17-gauge double-lumen needle (Cook Medical) and a vacuum pump (Cook Medical) under pressure at 125 mmHg. Each follicle sized above 12–14 mm was drained, and follicle flushing was not performed. The cumulus-oocyte complexes (COCs) were removed from the collection fluid using a sterile glass pipette and washed in G-IVF Plus media (Vitrolife, Sweden) and transported to the laboratory.

### Oocyte insemination and embryo culture

Oocytes were inseminated either by conventional IVF or by ICSI depending on sperm quality. Oocytes undergoing IVF insemination were placed into a dish with G-IVF (Vitrolife) covered in mineral oil. Oocytes undergoing ICSI were denuded and injected if maturation status was confirmed by the presence of the first polar body (PB). Fertilization was assessed 17–19 h after insemination and was defined by the presence of two pronuclears (2PN) and two PBs. All embryos were transferred to GM medium (G-M, Life Global, CT, USA) for a further 48 h of culture. Embryo development and quality were assessed 68–72 h (day 3) after insemination, based on the number of blastomeres, blastomere symmetry, percentage of fragmentation, and quality of cytoplasm according to the criteria established by the Istanbul Consensus Workshop on Embryo Assessment [26]. All supernumerary day-3 embryos were cryopreserved by vitrification (JIEYING laboratory Inc., Canada) for future use.

### Endometrial preparation and embryo transfer

All patients underwent transfer of day-3 embryos in a fresh cycle and subsequent frozen embryo transfer (FET) when the cryopreserved embryos generated from the index stimulation cycle were available. The embryos with the best morphological grade were selected for transfer. In absence of high-quality embryos, transfer of any embryo quality was considered after careful patient counselling.

In a fresh cycle, the luteal phase was supported with progesterone intravaginal gel (Crinone 8% 90 mg/day, Merck-Serono) commenced on the day of oocyte retrieval until 14 days after embryo transfer. In women with positive pregnancy test, luteal support was continued until 8 weeks gestation. The protocols used for FET utilized either natural cycle or artificial estradiol and progesterone endometrium priming in normo-ovulatory and oligo-ovulatory women, respectively. In natural cycle, ovulation was tracked with transvaginal ultrasound and urine LH kit. Oral dydrogesterone (Duphaston, 20 mg daily for 7 days; Abbott Biologicals B.V.) was commenced for luteal phase support 3 days after LH surge on the day of embryo transfer until 8 weeks gestation. In artificial FET protocol, oral estradiol valerate (Progynova 6 mg/day, Schering, Berlin, Germany) was initiated on the third day of the menstrual cycle and endometrial thickness was monitored with transvaginal ultrasonography. When the endometrial thickness exceeded 8 mm, luteal support with progesterone intravaginal gel (Crinone 8% 90 mg, daily; Merck-Serono), combined with oral dydrogesterone (Duphaston, 20 mg daily for 7 days; Abbott Biologicals B.V.) was added and embryo transfer was performed after 5 days. Hormonal treatment was stopped if pregnancy test was negative or continued until 11 weeks gestation with tapering off after 10 weeks. Single or double cleavage-stage embryo transfer were performed by using a soft catheter (K-Soft 5100; Cook, Queensland, Australia) without ultrasound guidance. Serum hCG was measured 14 days after embryo transfer and was considered positive for hCG level ≥ 10 IU. Transvaginal ultrasonography at 30 days after transfer was used to confirm clinical pregnancy.

### Hormone assay procedures

All the hormonal assays were performed at the endocrine laboratory of the Peking University Third Hospital Reproductive Centre by using commercially available kits. Serum concentrations of hCG were determined by using the commercially available ELISA kit (Beckman DXI800, Beckman, USA) according to the manufacturer’s instructions. Serum levels of anti-Mullerian hormone (AMH) were measured by automated assays using commercially available kit (Ashlab, USA). Serum luteinizing hormone (LH), FSH, estradiol (E2), and Progesterone (P) were tested using the Immulite 1000 assay based on chemiluminescence (DPC, Poway, CA).

The lower detection limit of the hCG and the AMH assays was 0.5 IU/L and 0.06 ng/ml respectively. The intra- and inter-assay coefficient of variation (CV) for hCG activity was 5% and for AMH was 8%. The lower detection limit of LH, FSH, E2 and P was 0.05 IU/L, 0.12 IU/L, 73.4 pmol/L, 0.64 nmol/L, respectively. The CV of LH and FSH was 6% and of E_2_ and P was 10%.

### Outcome measures

The primary outcome measure was the number of high quality day-3 embryos generated from one stimulation cycle. High quality embryos were defined as embryos that reached 6 to 8-cell stage with cytoplasmic fragmentation occupying less than 10% of the embryo surface and had equal size blastomeres.

The secondary outcomes included ovarian response parameters (duration of stimulation, total dose of gonadotrophins, peak E2 level and endometrial thickness on the day of hCG trigger), embryological parameters (number of oocytes retrieved, fertilization rate, number of patients with frozen embryos and number of patients who did not achieve embryo transfer) and clinical parameters (miscarriage, clinical pregnancy and live birth rate). Fertilization rate was defined as the number of 2PN embryos divided by the number of inseminated oocytes. Clinical pregnancy was defined as a presence of intrauterine gestational sac observed on ultrasound after 30 days of embryo transfer. Miscarriage was defined as a loss of clinical pregnancy before 24 weeks of gestation. Live birth was defined as the birth of at least one living child, irrespective of the duration of gestation. Clinical pregnancy and live birth rate were calculated per embryo transfer cycle as number of pregnancies/ live births divided per number of women who had transfer. Cumulative pregnancy and live birth rate were defined as the number of clinical pregnancies/live births generated from the index ART cycle following fresh or frozen embryo transfer divided by all women who received treatment. In addition, markers of ovarian reserve, including AMH, day 3 FSH and AFC were evaluated before and after CoQ10 treatment in the participants from the intervention (study) group.

### Sample size calculation

The sample size calculation for this study was based on the number of high quality embryos as primary outcome. In our center women with poor response have an average 0.6–0.8 high quality embryos per woman. To detect a difference of 50% in primary outcome measure (from 0.6–0.8 to 1.0–1.2 embryos per woman) with alpha 0.05 and power 0.80, the required sample size was estimated at 76 women in each arm. When accounted for a drop out rate of 20%, each arm required 92 women.

### Statistical analysis

The Student’s t-test or Mann-Whitney U test were used for comparisons of continuous variables between the groups depending on the distribution of the data. The chi-squared test or Fisher’s exact test, where appropriate, were used for comparisons of categorical variables. Results are presented as mean ± standard deviation (SD), median and interquartile range (IQR) or as percentages. Statistical significance was set at a probability (p) value < 0.05. All statistical analyses were performed using SPSS 22.0 software (IBM Corp., Armonk, NY, USA).

## Results

A total of 436 women met inclusion criteria. Of them, 186 women agreed to participate and were enrolled in the study, 93 women in each arm. Among the participants who were randomized to the intervention (CoQ10 treatment) group, 17 women were excluded from the analysis for the following reasons: one woman changed her mind to undergo ART and 16 women discontinued CoQ10 treatment due to the compliance issues. Overall, 76 women were retained in the study group and 93 women comprised the control group. All the participants shared the features of POSEIDON group 3, i.e. low prognosis patients younger than 35 years old with poor ovarian reserve pre-stimulation parameters. Baseline characteristics were comparable between the two groups with respect to age, BMI, duration of infertility, parity, ovarian reserve tests and causes of infertility (Table 1). Most participants were diagnosed with primary infertility and were ART treatment-naïve.

**Table 1:** Baseline characteristics of the study population

Variables	Study group (*n* = 76)	Control group (*n* = 93)	*p*-value
Demographic characteristics			
Age (years), mean ± SD	32.50 ± 3.30	31.92 ± 3.68	0.29
BMI (kg/m^2^), mean ± SD	21.85 ± 2.51	22.24 ± 3.07	0.37
Infertility duration (years), median (IQR)	3 (2, 4)	3 (2, 3)	0.32
Primary infertility, n (%)	48/76 (63.15)	65/93 (69.89)	0.71
Nulliparity, n (%)	72/76 (94.74)	86/93 (92.47)	0.76
Previous ART treatments, n (%)	11/76 (14.47)	20/93 (21.51)	0.43
Ovarian reserve markers			
AMH (ng/ml), median (IQR)	0.57 (0.35, 0.80)	0.56 (0.35, 0.80)	0.46
AFC, median (IQR)	5 (3, 6)	4 (3, 6)	0.17
Day 3 FSH (IU/ml), median (IQR)	12.25 (9.39, 15.50)	12.6 (9.95, 15.60)	0.58
Diagnosis of infertility in addition to POR			
Tubal factor, n (%)	13/76 (17.11)	22/93 (23.66)	0.61
Male factor, n (%)	22/76 (28.95)	25/93 (26.88)	0.82
Unexplained, n (%)	22/76 (28.95)	25/93 (26.88)	0.82

*AMH* - anti-Mullerian hormone; *AFC* - antral follicle count; *BMI* – body mass index; *IQR* – interquartile range; *POR* – poor ovarian reserve; *SD* – standard deviation

In the treatment group, no local or systemic side effects related to the use of oral CoQ10 were reported. Sequential measurements of ovarian reserve markers before and after CoQ10 treatment are presented in Table 2. The levels of basal day-3 FSH were significantly lower after 60 days supplementation of CoQ10 compared to the pre-treatment levels in the same group of women. In contrast, the levels of AMH and AFC were almost identical before and after CoQ10 treatment (Table 2).

**Table 2:** Ovarian reserve markers before and after CoQ10 treatment in the study group

Variables	Before CoQ10 (*n* = 76)	After CoQ10 (*n* = 76)	*p*-value
AMH (ng/ml), median (IQR)	0.57 (0.35, 0.80)	0.59 (0.38, 0.80)	0.91
AFC(n), median (IQR)	5 (3, 6)	5 (3, 7)	0.94
Day 3 FSH (IU/ml), median (IQR)	12.25 (9.39, 15.50)	10.50 (9.23, 12.60)	0.006

*AMH* - anti-Mullerian hormone; *AFC* - antral follicle count; *FSH* - follicle stimulating hormone; *IQR* - interquartile range

The parameters of ovarian response to stimulation and the embryology outcomes of ART cycles in the study population are summarized in Table 3. The amount of gonadotrophin used was significantly lower in CoQ10 treatment group than in controls (*p* = 0.03). The duration of gonadotrophin therapy tended to be shorter in the participants treated with CoQ10, but the difference did not reach statistical significance (*p* = 0.08). Peak E2 serum concentrations were significantly higher in the CoQ10 group, but there was no difference in the mean endometrial thickness on the day of hCG trigger between the two groups. In the CoQ10 treatment group there were fewer cancelled cases due to suboptimal ovarian response (5.23%, 4/76) compared to the control group (10.75%, 10/93) although this difference failed to achieve statistical significance, *p* = 0.27. Overall, 94.74% (72/76) women from the CoQ10 group and 89.25% (83/93) women from the control group received hCG and underwent oocyte retrieval. The median number of retrieved oocytes was significantly higher after CoQ10 pre-treatment (4, IQR 2–5), than in controls (2, IQR 1–2), *p* = 0.002. Most women had conventional IVF and the number of ICSI cycles was comparable between the groups. The median number of fertilized oocytes and fertilization rate were significantly higher in women treated with CoQ10 than in controls, *p* < 0.05. The median number of high quality day-3 embryos available per patient in the CoQ10 group was 1 (IQR 0–2) and in control group was 0 (IQR 0–1.75), with significant difference in favor of CoQ10 treatment, *p* = 0.03.

**Table 3:** ART cycle stimulation parameters and embryology outcomes

Variable	Study group (*n* = 76)	Control group (*n* = 93)	*p*- value
Cycle stimulation			
Total dose of Gn (IU), median (IQR)	2000 (1200, 4275)	3075 (1900, 4275)	0.03
Duration of stimulation (days), median (IQR)	10 (9, 11)	11 (9, 12)	0.08
Peak E2 concentration (pmol/l), median (IQR)	2349 (892, 4784)	1685 (1125, 3042)	0.02
Endometrial thickness on the day of hCG trigger (mm), mean ± SD	10.12 ± 1.93	10.34 ± 1.50	0.13
Patients who had oocyte retrieval	72/76 (94.74)	83/93 (89.25)	0.82
Cancelled cycles ^a^, n (%)	4/76 (5.23)	10/93 (10.75)	0.27
Embryology outcomes			
Retrieved oocytes, median (IQR)	4 (2, 5)	2 (1, 4)	0.002
ICSI cycles, n (%)	24/76 (31.58)	19/93 (20.43)	0.20
Fertilized oocytes (2PN), median (IQR)	0.80 (0.50, 0.93)	0.50 (0.33, 1.0)	0.01
Fertilization rate ^b^, n (%)	191/253 (67.49)	191/283 (45.06)	0.001
Number of high quality embryos, median (IQR)	1 (0, 2)	0 (0, 1.75)	0.03

^a^Included women in who did not respond to stimulation and did not have oocyte retrieval

^b^Calculated as following: the number of total 2PN embryos divided by the number of total inseminated oocytes

*E2* – estradiol; *Gn* – gonadotrophin; *hCG* – human chorionic gonadotrophin; *IQR* – interquartile range; *LH* - luteinizing hormone; *P* - progesterone, *2PN* – two pronuclear, *SD* - standard deviation

Among the patients in CoQ10 group who underwent oocyte retrieval, there was significantly lower number of women who did not achieve embryo transfer because of failure to retrieve oocytes or due to the absence of useable embryos (8.33%, 6/72) compared to women from the control group (22.89%, 19/83), *p* = 0.04 (Table 4). Collectively, embryos were available for 66 women in the CoQ10 group and 64 women in the control group, all of whom underwent fresh embryo transfer. The number of fresh embryo transfer cycles in the CoQ10 groups was comparable to that in controls. More patients in the CoQ10 group had cryopreserved embryos (18.42%, 14/76 vs. 4.3%, 4/93, respectively, *p* = 0.02) and the number of frozen-thaw embryo transfers from the index stimulation cycle was significantly higher, *p* = 0.01 (Table 4). In 14.29%, 2/14 women from the CoQ10 group with available cryopreserved embryos and in 25%, 1/4 controls, embryos did not recover after thawing. One to two embryos were replaced into the uterus in each transfer cycle with higher median number in the CoQ10 group (2, IQR 1–2) than in controls (1, IQR 1–2), *p* = 0.04.

**Table 4:** Clinical reproductive outcomes

Variable	Study group (*n* = 76)	Control group (*n* = 93)	*p*-value
Number of fresh ET cycles^a^, n (%)	66/76 (86.84)	64/93 (68.82)	0.35
Patients who had oocyte retrieval but no ET ^b^, n (%)	6/72 (8.33)	19/83 (22.89)	0.04
Number of FET cycles, n (%)	12/76 (15.79)	3/93 (3.23)	0.01
Patients with cryopreserved embryos, n (%)	14/76 (18.42)^c^	4/93 (4.30)^d^	0.012
Number of embryos per ET^e^, median (IQR)	2 (1,2)	1 (1,2)	0.04
Clinical pregnancy rate per fresh ET^f^, n (%)	23/66 (34.85)	16/64 (25.00)	0.24
Cumulative clinical pregnancy rate^g^, n (%)	24/76 (31.58)	16/93 (17.20)	0.11
Multiple pregnancy, n (%)	4/76 (5.26)	3/93 (3.23)	0.70
Spontaneous miscarriage, n (%)	2/23 (8.67)	2/16 (12.50)	0.73
Live birth rate per fresh ET^f^, n (%)	21/66 (31.82)	14/64 (21.88)	0.33
Cumulative live birth rate^g^, n (%)	22/76 (28.95)	14/93 (15.54)	0.08

^a^All patients with available embryos had fresh ET

^b^Included women who had hCG andoocyte retrieval but did not have oocytes or useable embryos

^c^Embryos for 2women from this group did not survive the thawing (2/14, 14.29%)

^d^Embryos for 1 woman from this group did not survive the thawing (1/4, 25%)

^e^All the transferred embryos were day-3 cleavage stage embryos

^f^Calculated as follows: the number of clinical pregnancies/ live births originated from fresh ET divided by the number of women with transferred embryos

^g^Calculated as follows: the number of clinical pregnancies/ live births originated from one completed ART cycle including fresh and frozen-thaw ETs divided by the number of women treated

*ET* – embryo transfer; *FET* – frozen-thaw embryo transfer; *IQR* – interquartile range

In the CoQ10 group there were 23 clinical pregnancies following fresh embryo transfer and one additional pregnancy following frozen-thaw embryo transfer. In the control group there were 16 clinical pregnancies after fresh embryo transfer and no pregnancies after frozen-thaw transfer. The were no spontaneously conceived pregnancies in either group. Successful live birth was achieved in 22 women from the CoQ10 (21 after fresh and 1 after frozen-thaw transfer) and in 14 women from the control group. Clinical pregnancy rate and live birth rate per fresh embryo transfer cycle were 34.85% and 31.82% in women treated with CoQ10, and 25% and 21.88% in controls, respectively. The clinical estimates for frozen-thaw embryo transfer were not calculated due to the paucity of the available data. When the transfers of all embryos originating from the complete ART cycle were considered, in women treated with CoQ10 the cumulative clinical pregnancy rate after one complete cycle was 31.58%, 24/76 and the cumulative live birth rate was 28.95%, 22/76. In the control group, the cumulative clinical pregnancy rate was 17.20%, 16/93 and the cumulative live birth rate was 15.54%, 14/93, respectively. Miscarriage rate was 8.67% in women from the CoQ10 group and 12.5% in controls. Although women from the CoQ10 group had higher clinical pregnancy and live birth rates with lower occurrence of pregnancy loss, the difference between the treatment and control groups failed to achieve statistical significance for each of these outcomes.

## Discussion

In this study we demonstrated potential benefit of CoQ10 treatment in improving ovarian response to gonadotrophin stimulation in young women with low ovarian reserve. To the best of our knowledge, this is the first study that evaluated an effect of anti-oxidant treatment in specific phenotypic subgroup of women with POR.

Our results demonstrate that pre-treatment with CoQ10 resulted in significant decrease in the total amount of gonadotrophin needed to achieve ovarian response, shorter duration of stimulation, higher peak E2 levels and the number of oocytes retrieved. CoQ10 treatment led to significant increase in fertilization rate and in the number of high quality embryos. There was significantly lower rate of cancelled cycles because of no response to stimulation, less cancelled embryo transfers because of failed embryo development and larger number of cycles with cryopreserved embryos in the CoQ10 treated group than in controls. The clinical pregnancy and live birth rates were higher after CoQ10 treatment then in controls, but these differences failed to achieve significance, presumably due to insufficient sample size. Taken together, our data suggest that CoQ10 administration enhances ovarian response to stimulation and improves oocyte and embryo quality.

The findings of this study are approximately in line with previous reports that linked CoQ10 with improved reproductive outcomes. Small randomized placebo-controlled study in 24 participants (10 women in CoQ10 and 14 in placebo group) have demonstrated higher peak concentration of E2, increased number of high quality cleavage embryos, and a trend towards decreased aneuploidy and higher clinical pregnancy rate after 60 days treatment with 600 mg CoQ10 [25]. However, the study was underpowered and failed to demonstrate significant difference in clinical outcomes between the groups [25]. Another randomized controlled study in 101 young women with PCOS demonstrated that addition of CoQ10 in a dose of 180 mg during ovulation induction with clomiphene citrate improved ovarian response in clomiphene-resistant women and resulted in higher clinical pregnancy rate [24]. Retrospective analysis in 797 IUI and 253 IVF cycles in women older than 36–37 years revealed that addition of 600 mg CoQ10 to dehydroepiandrosterone (DHEA) over the period longer than 1 month resulted in lower dose of gonadotrophins and higher number of mature follicles than in women treated with DHEA alone [27]. The authors did not demonstrate significant difference in the embryological or clinical outcomes, and the comparisons with untreated controls were not available [27].

The plausible effect of CoQ10 on reproductive function is attributed to its effect on the antioxidative capacity and energy production in the oocyte [10, 28, 29]. CoQ10, the only synthesized lipid soluble antioxidant in humans, is an essential component of the mitochondrial respiratory chain, serving an important antioxidant function both in mitochondria and in lipid membranes [15]. ROS-induced DNA damage in ovary leads to genomic instability, mutations and apoptosis of oocytes, and is thought to be ameliorated by an antioxidant activity of CoQ10 [22]. CoQ10 has been also shown to improve mitochondrial function and restore energy production by mitochondria [23]. Mitochondrial dysfunction in oocytes results in decreased oxidative phosphorylation and suboptimal levels of mitochondria-generated ATP, which has been strongly associated with poor reproductive performance, including diminished ovarian reserve, poor oocyte quality, abnormal fertilization and deranged preimplantation embryo development [29, 30]. Energy production by mitochondria is important for steroid hormone biosynthesis, oocyte maturation, fertilization, and early embryonic development [31, 32]. It has been demonstrated that CoQ10 supplemented in aged animal model has improved mitochondrial membrane potential, mitochondrial ATP production and mitotic spindle orientation [21]. Treatment with CoQ10 increased the number of ovulated oocytes and reduced ROS in oocytes to the levels observed in young animals, indicating this is an effective strategy to reverse the effect of reproductive ageing [33]. In humans, levels of CoQ10 in the follicular fluid positively correlated with oocyte maturation, embryo grade and pregnancy rate in women undergoing ART [34, 35]. While oocyte appears to be the main target of CoQ10, it remains unclear whether anti-oxidant treatment also improves uterine environment. We did not demonstrate any differences in endometrial thickness, but there were no data to confidently comment on the effect of CoQ10 in intra-uterine milieu.

CoQ10 has been also associated with improved ovarian reserve. In rodents, CoQ10 administration reversed ovarian toxicity of cisplatin, leading to increase in the serum AMH concentrations, improved AMH-positive follicle count and lower number of atretic follicles [22]. Exposure to CoQ10 restored ovarian reserve in mice with induced accelerated oocyte loss [21]. Currently, however, there is relative paucity of information concerning the exact mechanism by which CoQ10 influences ovarian reserve in humans and it is difficult to conclude whether CoQ10 rescues follicles from apoptosis or enhances primordial follicle activation. In this study there was significant decrease in baseline FSH levels after 60 days of CoQ10 administration. It is possible that a change in FSH levels could also have occurred without CoQ10 treatment over a period of two-three months, but this seems unlikely considering that previous study in 287 infertile men showed 14% decline in FSH levels after 3 months of CoQ10 supplementation with continuing decrease throughout 12 months therapy [18]. In contrast, we did not observe improvement in other ovarian reserve markers, namely AMH and AFC and such discrepancy between our and animal studies could be explained by different treatment protocols and variation in physiological parameters between species. In rodents, 8–12 weeks of CoQ10 exposure corresponds to about ¼ of the life span, which is considerably longer interval in relation to a reproductive cycle when compared to analogous treatment period in humans. It has been supposed that two months exposure to CoQ10 could improve energy production in the ovary but might not be long enough to restore prolonged effect of oxidative damage [25]. It should be noted that it takes about three months for a primordial follicle to reach the preovulatory stage [36]. AMH is predominantly produced upon transition from the primordial to primary follicles when they are recruited from the dormant pool and represents early stages of growth [37]. Thus, short duration of CoQ10 administration is likely to influence late events of follicle maturation but may not be sufficient to improve follicle recruitment evidenced by AMH levels. Indeed, the study that reported significant increase in antral follicles in CoQ10, included women who were treated with CoQ10 for an average of 8.8 ± 6.2 months [27].

The optimal timing, duration and dose of CoQ10 supplementation remain unclear. In this study, the duration of treatment was selected arbitrary based on previous study in IVF population [25]. It could be argued that CoQ10 treatment implies a delay in initiation of ART cycle and thus longer pretreatment period may be less acceptable to the patients. It has been demonstrated that CoQ10 is well tolerated and safe for healthy adults at intake of up to 900 mg/day [38]. We were guided by previous experience in selecting the dose of CoQ10, although this was rather intuitive choice [18, 24, 25, 27].

The main strength of this study is that it focused on a specific phenotype within a broad heterogeneous group of women with POR. All the participants shared similar demographic and clinical characteristics and had comparable pre-treatment markers of ovarian activity. In addition, we utilized an unbiased randomization process and applied the similar laboratory and clinical protocols to all the participants.

The important limitation of our study was its small sample size and we were unable to detect significant differences in clinical outcomes. Live birth is an ultimate outcome of infertility treatment and is more appropriate estimate for patient counseling. The POSEIDON group has recently suggested that the number of oocytes needed to obtain at least one euploid embryo per patient is a more practical treatment endpoint for the studies in women with POR and helps to define the short-term goals for management [10]. In adopting this approach, we chose the number of high quality embryos as a primary outcome measure and calculated the sample size accordingly. High drop-out rate in the study group due to CoQ10 discontinuation was additional limiting factor that should be considered in future studies. In line with the reported by others, CoQ10 administration did not cause any adverse reactions or side effects in this study [39], but all women who discontinued treatment reported difficulty to comply with the CoQ10 regime requiring three times a day administration. Finally, in this study we did not evaluate the levels of oxidative stress markers before or after treatment and did not assess the influence of other lifestyle factors that may pose women at higher risk. A threshold effect of CoQ10 may vary on individual level due to interference with other environmental exposures leading to oxidative stress and this should be considered in the design of future studies.

## Conclusions

In summary, pretreatment with CoQ10 increases ovarian response to stimulation and improves oocyte and embryo quality in young low prognosis patients with diminished ovarian reserve. There is a possible beneficial effect on clinical pregnancy and live birth rates, but this needs to be confirmed in larger randomized controlled studies. Further work is required to establish the optimal length, timing and dosage of treatment and to evaluate the therapeutic effect of CoQ10 supplementation in other subgroups of low prognosis women with POR.
